# Composite Hydrogels with Rapid Self-Healing, Stretchable, Moldable and Antibacterial Properties Based on PVA/ε-Poly-l-lysine/Hyaluronic Acid

**DOI:** 10.3390/molecules29194666

**Published:** 2024-09-30

**Authors:** Na Sun, Xiangnan Liu, Wenqi Lv, Chunlin Xu, Ailing Zhang, Panpan Sun

**Affiliations:** 1 School of Bioscience and Technology, Shandong Second Medical University, Weifang 261053, China; 17852209733@163.com (X.L.); 15662573685@163.com (W.L.); xuchunlin2024@163.com (C.X.); 2 College of Pharmacy, Shandong Second Medical University, Weifang 261053, China; sunna@sdsmu.edu.cn; 3 College of Chemical Engineering and Environmental Chemistry, Weifang University, Weifang 261061, China

**Keywords:** composite hydrogels, self-healing, antibacterial activity

## Abstract

Self-healing, stretchable, and moldable hydrogels have a great potential application in tissue engineering and soft robotics. Despite great success in reported hydrogels, it is still a great challenge to construct the moldable hydrogels with an ultrafast self-healing performance. Herein, the composite hydrogels (PBLH) with ultrafast self-healing, stretchable, and moldable properties were successfully constructed by poly (vinyl alcohol) (PVA), borate (B), ε-poly-l-lysine (EPL), and hyaluronic acid (HA) based on an efficient one-pot method. Fourier transform infrared spectroscopy, X-ray diffraction, and rheological measurements confirmed the formation of a dynamic network among PVA, B, EPL, and HA through the cross-linking of dynamic borate bonds, electrostatic interaction, and hydrogen bonding. Having fabricated the dynamic network structure, the damage gap of the composite hydrogels can heal within 1 min, presenting an excellent self-healing ability. Simultaneously, the composite hydrogels can be molded into various shapes, and the length of the composite hydrogels can be stretched to 15 times their original length. In addition, the composite hydrogels exhibited an excellent antibacterial property against *Staphylococcus aureus* (*S. aureus*) and *Escherichia coli* (*E. coli*). Our results illustrated that the composite hydrogels not only retain the advantages of traditional hydrogels but also possess ultrafast self-healing, outstanding stretchable and antibacterial properties, presenting a prospective candidate for constructing biomedical materials.

## 1. Introduction

Hydrogels are unique macromolecular materials with high water content and unique three-dimensional networks [1], presenting an extensive application in the fields of wound dressing [2], tissue engineering scaffolds [3], wearable electronics [4], drug delivery [5,6], and so forth. Such applications are attributed to their multiple properties, such as self-healing, stretchability, moldability, compressibility, and flexibility. Among these properties, the self-healing ability has become a research hotspot [7,8,9]. Self-healing property is quite common in living creatures. An organism can automatically repair damages by activating the self-healing process, especially of human skin. In recent years, more and more self-healing hydrogels have been exploited to avoid structural deterioration and loss of components, thus prolonging the life span of material. To date, the polymers, such as PVA [10,11,12,13], hyaluronic acid (HA) [14,15], chitosan [16], and agarose (Agr) [17], are common to construct self-healing hydrogels due to their outstanding biocompatibility, nontoxicity, accessibility, and tunable physical properties. Generally, chemical cross-linking and non-covalent combining are the main patterns for fabricating self-healing hydrogels. The specific approaches consist of dynamic covalent bonds (e.g., imine, acylhydrazone, borate ester, disulfide, etc.) [18,19,20], host–guest interaction (e.g., β–cyclodextrin) [21], and physical interactions (e.g., hydrogen bonding, ionic bonding, etc.) [22,23]. However, the mechanical strength and self-healing ability of the traditional single-network hydrogels are generally poor, which somewhat hinders the development of self–healing hydrogels for practical applications. To enhance the mechanical property and self-healing ability of hydrogels, the composite hydrogels have been exploited due to their controllable mechanical performance.

In recent years, composite hydrogels have attracted considerable attention in self-healing material fields. Composite hydrogels, generally consisting of at least two intertwined crosslinked building blocks (polymers or networks), can be prepared via different methods, such as chemically–chemically crosslinked, physically–chemically hybridized, and physically–physically crosslinked [24,25,26,27,28,29,30,31]. However, chemical crosslinking generally refers to the multi–step synthetic method, which is complicated and time–consuming and also uncontrollable. The hydrogel was fabricated by physical crosslinking, including electrostatic interactions, van der Waals force, hydrogen bond binding, and host–guest interactions, which were easily damaged due to the physically crosslinked structures with conformational changes. The composite hydrogels, constructed by dynamic covalent bonds and physical interactions, demonstrate extraordinary physical and mechanical properties [32,33]. Generally, one of the building blocks for constructing composite hydrogels is using the cross-linking reaction between PVA and borate. Note that the building block is fabricated by the formation of dynamic borate ester bonds between two diol units and borate ions. For instance, Jiang et al. fabricated an ultrafast self-healing hydrogel by cross-linking agarose/PVA/borax composite double networks through dynamic boron ester bonds [34]. Dong et al. developed a highly stretchable and self-repairing hydrogel based on PVA/borax, coupled with the hydrogen bonding of carboxymethyl cellulose sodium (CMC) [2], which significantly accelerated wound healing by reducing bacterial infections. Recently, antibacterial agents as another network were introduced to the soft PVA/borate network and then constructing antibacterial composite hydrogels. Wang et al. designed the highly stretchable, moldable, rapid self-healing composite hydrogels with good antioxidant and antibacterial properties based on embedding CNF and TA into PVA and borax hydrogel networks, which provided a facile approach to fabricate a kind of multifunctional composite hydrogels [35].

Recently, cationic polypeptides ε-poly-l-lysine (EPL), containing 25–30 lysines, have been applied to develop antibacterial materials, wearable materials, drug/gene carriers, and so on, owing to their controllable antibacterial properties and high biocompatibility [36,37]. For example, Kim et al. reported a method for generating multinucleated colonies by chemically modifying single–walled carbon nanotubes via poly-l-lysine [38]. In addition, a photocurable hydrogel based on ε-poly-l-lysine (EPL) composite was fabricated in situ by photocuring crosslinking reaction using glycidyl methacrylate and then complexed with tannic acid (TA) to improve the mechanical stability and antibacterial performance of the EPL hydrogels [39]. However, the chemical modifications of EPL derivatives generally involve multiple and complex chemical synthesis steps, which limit their application in constructing the antibacterial materials.

**Scheme 1:**
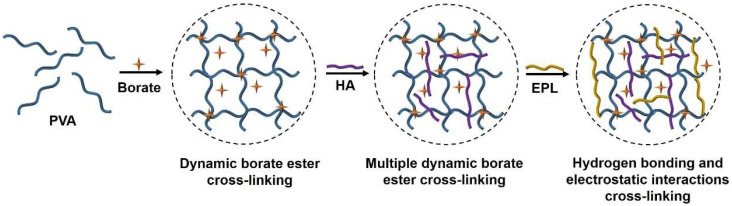
Schematic diagram of the mechanism of composite dynamic network hydrogel through dynamic borate eater reaction and physical cross-linking.

In this work, we successfully fabricated the composite hydrogels based on PVA, borate, EPL, and HA, with the introduction of EPL into the network just via simple mixing. As shown in Scheme 1, one of the building blocks was formed by PVA, borate, and HA through multiple dynamic borate eater cross–linking. Another building block was fabricated by forming strong hydrogen bonding and electrostatic interactions, for which the EPL acted as a cross–linker. The intertwined of the two building blocks not only enhanced the mechanical strength of composite hydrogels but also endowed them with excellent self–healing, stretchable, and moldable properties. Moreover, these properties and the rheological behaviors and morphology of composite hydrogels were evaluated. Simultaneously, the antibacterial performance of the formed hydrogels was assessed by monitoring the viability of *S. aureus* and *E. coli* under scrutiny. All the findings indicate that the composite dynamic hydrogel with multiple properties paves a promising prospect for biomedical materials.

## 2. Results

### 2.1. Fabrication of the Composite Hydrogels

**Figure 1:**
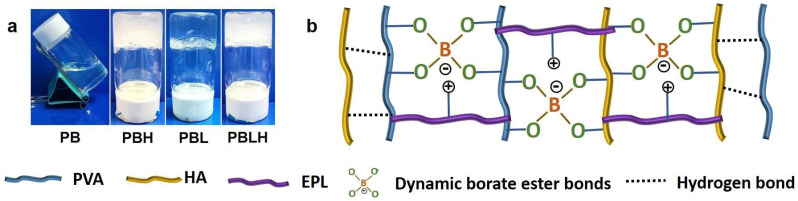
(**a**) The photographs of the states of the hydrogels, PB (10.0 wt% PVA and 0.5 wt% borax), PBH (10.0 wt% PVA, 0.5 wt% borax and 4.0 wt% HA), PBL (10.0 wt% PVA, 0.5 wt% borax and 0.5 wt% EPL), and PBLH (10.0 wt% PVA, 0.5 wt% borax, 4.0 wt% HA, and 0.5 wt% EPL). (**b**) Schematic illustration of the interactions of composite dynamic network hydrogels between all of the components, including the dynamic borate eater reaction and physical cross–linking.

Recently, the composite hydrogels have drawn more and more attention due to their outstanding mechanical property and self–healing ability. In this study, composite dynamic network hydrogels were successfully fabricated by employing the PVA, HA, borate, and EPL through the one–pot method. The borate, EPL, and HA were introduced into the colorless and transparent PVA solutions with a certain concentration under agitation and heating, and then the pearl white composite hydrogels formed. The resulting composite hydrogels were confirmed via the inverted–tube method [40], as shown in Figure 1a. One of the building blocks was formed by cross–linking between PVA and borate. Borate, acting as a cross–linking agent, could react with the diol structure of PVA and further form dynamic covalent borate bonds. We systematically investigated the states of the formed hydrogels with different compositions, founding PB, PBH, and PBL hydrogels (the composition of these hydrogels illustrated in Figure 1) flowed after inversion for 1 h, as depicted in Figure S1. This phenomenon illustrated these hydrogel systems with weak mechanical properties due to the invertible nature of the borate bond and the weak hydrogen bond, which the chain of PVA and HA or EPL in the hydrogels can still move. The weak mechanical properties of these hydrogels restricted their applications in the fields of biological medicine. Thus, incorporating another building block may be a promising strategy to resolve these limitations. In this system, EPL was employed as a candidate to fabricate the second building block. The introduced EPL crosslinked with the original building block (PBL) via hydrogen bonds and electrostatic interaction (Figure 1b), resulting in the two building blocks intertwined, and then the composite hydrogels (PBLH) formed. The intermolecular hydrogen bonds are formed between the –NH_2_, –NH– groups of EPL, the –O– group of PVA, and the –NH–, –OH, and C=O groups of HA. The electrostatic interactions existed in the –NH_3_^+^ of EPL and the B(OH)_4_^−^ of borate or COO^−^ of HA. Notably, the formed PBLH hydrogels did not flow under the inverted tube even for 12 h. It is demonstrated that the addition of the second building block endows the composite hydrogels with strong mechanical properties and structures [35].

### 2.2. Structure and Micromorphology of Composite Hydrogels

**Figure 2:**
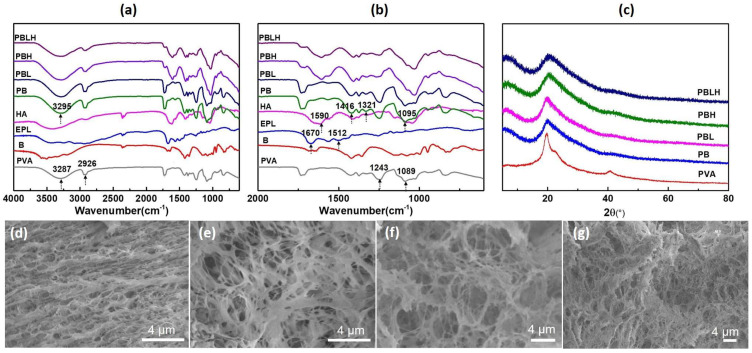
(**a**,**b**) The FTIR spectra of pure PVA, B, EPL, HA, and the formed hydrogels of PB, PBL, PBH, and PBLH. (**c**) XRD patterns of PVA, PB, PBL, PBH, and PBLH. (**d**–**g**) SEM images of PB, PBL, PBH, and PBLH, respectively. Scale bar = 4 μm.

Fourier transform infrared spectroscopy (FTIR) was employed to investigate the functional groups and the interactions among the formed hydrogels. As shown in Figure 2, the characteristic peaks and changes in PVA, borate, HA, EPL, and the resulting hydrogels in the spectra were illustrated. For neat PVA, the prominent absorption bands at around 1089, 2926, and 3287 cm^−1^ correspond to the stretching vibrations of C–O, C–H, and O–H, respectively [35]. In the spectrum of the EPL sample, the stretching vibrations of typical amide I and II absorption bands around appeared at 1670 and 1512 cm^−1^ [3]. Moreover, the band at 1590 cm^−1^ represents the C=O stretching in the spectrum of HA. After crosslinking with borate (PB), it was observed that the red–shift of the stretching vibrations of C–O and O–H to 1095 and 3295 cm^−1^. Furthermore, two new characteristic peaks at 1321 and 1416 cm^−1^ appeared, which were attributed to the asymmetric stretching relaxation of B–O–C [34]. These results were strong evidence for confirming the formation of the dynamic borate ester bonds between borate and PVA chains. Clearly, after introduction of EPL and HA into the PB system, the typical amide I and II absorption bands shifted to lower wavenumbers. As we all know, the intra- or intermolecular hydrogen bonding could reduce the force constants of the chemical bonds and result in their vibrational frequencies shifting to lower wavenumbers [3]. Hence, these blue–shifts of the absorption bands proved the formation of H-bonding between EPL and HA.

X-ray diffraction (XRD) is a powerful tool for analyzing and identifying the crystalline and amorphous phases of materials. The sharp diffraction peaks represent a high degree of crystallinity, while broad diffraction peaks indicate a low degree of crystallinity. To explore the crystal structure changes before and after gelation, XRD diffraction patterns of hydrogels were employed. Obviously, as illustrated in Figure 2c, the XRD patterns of PVA have three diffraction peaks at 2θ = 19.6°, 2θ = 22.9°, and 2θ = 40.8°, which correspond to the (101), (200), and (103) planes of PVA crystallites [36]. The stable crystal structures of PVA attribute to the PVA molecule containing a large number of hydroxyls as hydrogen bond sites. After crosslinking with borate (PB), the sharp diffraction peak at 2θ = 19.6° turned into a blunt peak, and the diffraction peaks at 2θ = 19.6° and 2θ = 22.9° disappeared. The changes in the specific peaks of PVA crystallites are probably attributed to the strong interactions between the −OH group of PVA and borate, which led to the destruction of PVA crystallite structures [3,35]. Compared with the PB system, the typical diffraction peaks of PBL, PBH, and PBLH hydrogels changed slightly with the addition of EPL and HA, further confirming the formation of the strong interactions between PVA, B, EPL, and HA during the cross–linking and intertwinement process [35].

The cross–sectional microstructures of PB, PBL, PBH, and PBLH hydrogels were characterized by using SEM, as shown in Figure 2d–g. Apparently, the differences in cross-linking networks and pore sizes of the dried hydrogels can be observed. The PB system shows well-defined denser lines and weak networks, which illustrated that the microstructures of PB may be branched polymers instead of networks. Compared with PB, a lot of larger pores emerge in the appearance of PBL or PBH hydrogels, while PBLH hydrogels with both EPL and HA exhibit a tighter network with thicker walls and smaller pores, which may attribute to the increased degree of cross–linking in composite network hydrogel. Thus, the incorporation of the EPL and HA could form an additional network and further intertwine with the before networks, which is beneficial to enhance the mechanical and performance of the composite hydrogels.

### 2.3. Rheology and Self-Healing Properties of the Composite Hydrogels

**Figure 3:**
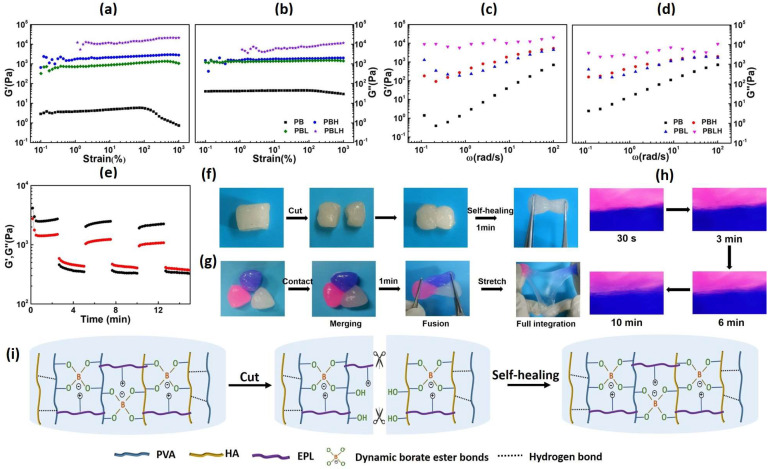
(**a**,**b**) Dynamic oscillatory strain sweep tests (from 0.1% to 1000%) and (**c**,**d**) frequency sweep tests (from 0.1 rad/s to 100 rad/s) of the hydrogels. (**e**) G′ and G″ of the PBLH hydrogels under alternate low (2%) and high (100%) strain at a fixed frequency of 1%; images of the in situ (**f**) (unstained hydrogels) and non-in situ (**g**) (stained hydrogels) methods were used to observe self-healing properties; the hydrogels were stained by methylene blue and eosin, respectively. (**h**) Photographs of the self-healing process of PBLH hydrogels by microscope. (**i**) Schematic diagram of the self-healing mechanism of PBLH hydrogels.

The mechanical properties of hydrogels play an important role in determining their suitability for specific applications. Rheology measurements are usually used to characterize the performance of hydrogels. In the rheological diagram, the storage modulus (G′) reflects the energy storage, which represents the elastic properties (solid–like behavior) of samples, while the loss modulus (G″) reflects the energy loss, which represents the viscous properties (liquid–like behavior) of samples [41]. As can be seen from Figure 3a,b, the G′ of PB, PBL, and PBH were lower than G″ over the entire region of strain testes, showing the Newtonian–like flow character, which demonstrated the formed structures may be branched polymers rather than networks. However, the PBLH hydrogels exhibited the typical solid-like character (G′ > G″) over the entire region of strain tests, illustrating the formation of networks in PBLH hydrogels. Simultaneously, the values of G′ and G″ were higher than those of PB, PBL, and PBH, indicating the mechanical property of PBLH hydrogels was enhanced by the combination of branched polymers with networks [15], which confirmed the formation of the composite network. In addition, in the frequency sweep test (Figure 3c,d), both G′ and G″ for all of the hydrogels gradually improved with frequency increasing, exhibiting frequency–dependent behavior. As was reported, the dynamically crosslinked hydrogels generally demonstrate frequency–dependent modulus, while the permanently crosslinked hydrogels show frequency–independent modulus [42,43]. Therefore, through analysis of the rheological studies, the network of the composite hydrogels is dynamic. Furthermore, the value of G′ is always bigger than that of G″ over the frequency test range, and the gelation state can be maintained within the range of 0.1–100 rad/s.

Generally, the dynamic nature of networks usually endows the materials with dynamic performance. We systematically investigated the self–healing behaviors of the PBLH hydrogels by macroscopic and rheological recovery tests. Initially, the self-repairing ability was measured by using the rheological test in different strains (2–100%-2–100%-2–100%). As illustrated in Figure 3e, when the strain is at 2% low strain, there is no apparent change in the values of G′ and G″, and the typical solid-like properties of hydrogels could be maintained (G′ > G″), while the networks of hydrogels are collapsed at the 100% high strain (G′ < G″). As the strain is restored to 2%, the G′ and G″ of the PBLH hydrogel instantly recover to the initial values, revealing the hydrogel network structures were reconstructed [44]. Noticeably, the values of G′ and G″ nearly returned to the original values even after three interval oscillatory strain switching back and forth tests, demonstrating the excellent self-healing ability of PBLH hydrogels.

Subsequently, we further researched the self–healing behaviors of PBLH hydrogels by in situ and non–in situ self–healing tests. As shown in Figure 3f, two pieces of unstained PBLH hydrogel contacted each other, and the fractured hydrogels immediately healed into a whole after 1 min without any external force. The reason for the self–healing is due to the dynamic borate ester bonds between the –OH of PVA or HA and borate. In addition, the two stained hydrogels and unstained hydrogel were gently put together, and then the three different hydrogels integrated into a new hybrid hydrogel rapidly. This hybrid hydrogel could maintain its integrity even under stretching and picking up with tweezers. Surprisingly, the hybrid hydrogel could be stretched into a membrane, indicating fast ex situ self–healing performance of PBLH hydrogel. The self–healing mechanism of the complete merging is explained by a sufficient amount of free –OH, –NH_2_, –NH_3_^+^, and borate on the surface of the hydrogels. They tend to create borate ester bonds, hydrogen bonds, and electronic interactions at the contacted interface [39]. Furthermore, the self–healing processes of the healing surface between two stained hydrogels were further recorded by optical microscopy (Figure 3h and Figure S2). Apparently, with the passage of time, the gap between the two stained hydrogels decreased and even completely disappeared after 10 min. Simultaneously, the dye molecules continuously spread across the fusion interfaces during the healing process and eventually interacted with each other at the boundary, testifying the dynamic networks of hydrogels [45]. Based on the overall results, we suspected that the extremely fast self–healing property of PBLH hydrogels may be attributed to the synergistic effect of the dual dynamically reversible borate diol bonds, electronic interaction, and hydrogen bonds (Figure 3i).

### 2.4. Stretchable, Shapeable, and Adhesive Properties of PBLH Composite Hydrogels

**Figure 4:**
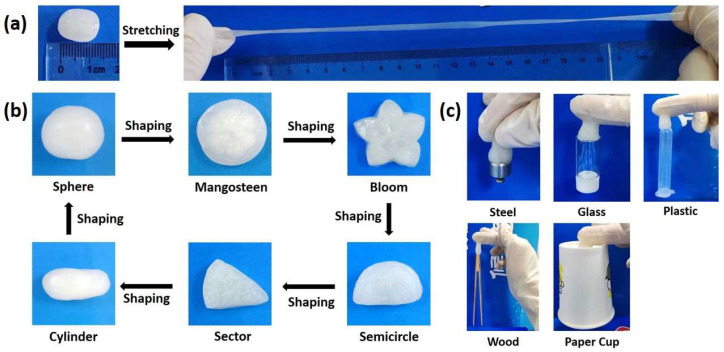
Photographs of the stretchable (**a**) shapeable (**b**) and adhesive properties (**c**) of the PBLH composite hydrogels.

The suitable stretchable, shapeable, and adhesive properties of materials are necessary to meet the requirements of different wounds. Herein, the stretchable, shapeable, and adhesive performance of the PBLH hydrogels were evaluated by macroscopic tests. Interestingly, the composite PBLH hydrogels exhibited a high stretchable ability, which could be stretched to more than 15 times of that original length, and without any visible crack appeared even under twisting (Figure 4a and Figure S3). Moreover, as can be seen from Figure 4b, the obtained composite PBLH hydrogels could be easily molded and remolded into a variety of shapes, including sphere, mangosteen, bloom, semicircle, sector, and cylinder, suggesting hydrogels with outstanding ductility and shapeable ability. In addition, the hydrogels exhibited good adhesion to the various different characters of objects, such as steel, glass, plastic, wood, and paper cups. Overall, the excellent adjustable properties of PBLH composite hydrogels exhibited potential applications for biomedical engineering, such as wound dressings.

### 2.5. Antibacterial Activity of the Hydrogels

**Figure 5:**
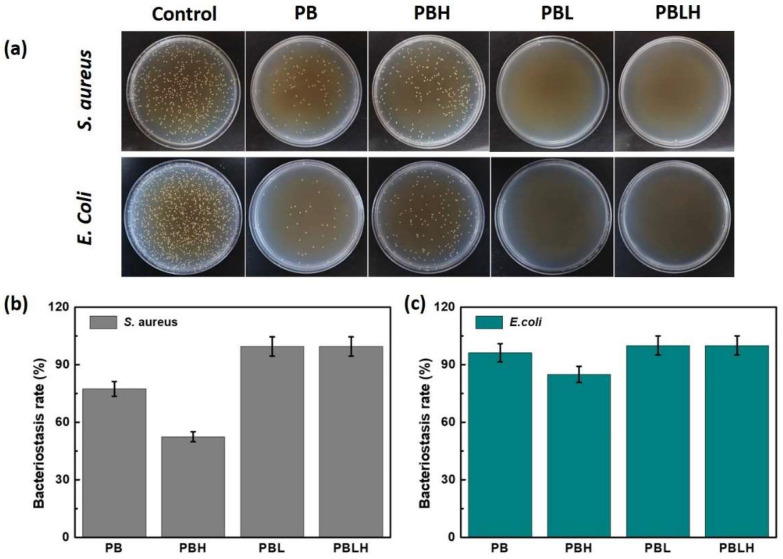
Digital photos of the growth of *S. aureus* and *E. coli* cultured in vitro on agar plates under different conditions (**a**). Bacteriostasis rate against *S. aureus* (**b**) and *E. coli* (**c**).

To evaluate the antibacterial performance of the fabricated composite hydrogels, *S. aureus* and *E. coli* were selected as the test bacteria. As shown in Figure 5a, in comparison with the control group, only a few or even none of the bacteria survived from the agar plate tests treated by the composite hydrogels, suggesting the hydrogels had outstanding antibacterial properties. Obviously, from the column chart of bacteriostasis rate (Figure 5b,c), the hydrogels show lower antibacterial activity against Gram-positive *S. aureus* than Gram-negative *E. coli.* In addition, the antibacterial performance of composite hydrogels was enhanced by the incorporation of the EPL. These results may attribute to the –NH and –NH_2_ groups of EPL, which can attach to the surface of bacteria and then destroy their cell membrane.

## 3. Materials and Methods

### 3.1. Materials

Poly(vinyl alcohol) (PVA, alcoholysis: 85.0–90.0 mol%, viscosity: 20.0–30.0 mPa), Hyaluronic acid (HA, molecular weight 80–100 KDa), and ε-poly-l-lysine (EPL, 98%, *n* = 25–35) were supplied by Tianjin Xiensi Biochemical Technology Co., Ltd. (Tianjin, China). Borate (B, sodium tetraborate decahydrate, 99.5%) was purchased from Sinopharm Group Chemical Reagent Co., Ltd. (Shanghai, China). All the materials were used as received without any purification. Deionized water was used throughout all the experiments.

### 3.2. Hydrogel Preparation

The hydrogels were prepared by using the one–pot method; the details of the preparation process are as follows: A certain amount of PVA was dissolved in hot deionized water and agitated at 95 °C for a duration of 2 h, resulting in the formation of PVA solutions with a concentration of 10 wt%. Then, some amount of borate, HA, and EPL were mixed in an aqueous solution and agitated until the solid dissolved thoroughly. Finally, the white PBLH hydrogels were obtained. With the same preparation steps, the hydrogels of PB, PBL, and PBH were obtained. The ingredients of these hydrogels are illustrated as follows:

PB: 10.0 wt% PVA and 0.5 wt% borax;
PBL: 10.0 wt% PVA, 0.5 wt% borax, and 0.5 wt% EPL;
PBH: 10.0 wt% PVA, 0.5 wt% borax, and 4.0 wt% HA;
PBLH: 10.0 wt% PVA, 0.5 wt% borax, 4.0 wt% HA, and 0.5 wt% EPL.

### 3.3. Characterization

Fourier Transform Infrared Spectroscopy (FTIR). FTIR spectra of hydrogels were obtained by using a Thermo Fisher Spectrum over a range of 4000–400 cm^−1^ with a resolution of 4 cm^−1^ d and a total of 32 scans for each sample.

Rheological Measurements. The rheological properties of the composite hydrogels were assessed using an Anton–Paar rheometer. A cone–plate system of C35/1° Ti L07116 with a plate diameter of 35 mm and cone angle of 1°. All rheological tests were carried out at 25.0 °C. In dynamic oscillatory strain sweeping tests, fixing frequency at 1 Hz, the strain sweeps over a range rate from 1% to 1000%. In frequency sweeping tests, fixing the shear rate at 1%, the frequency sweeping from 0.01 Hz to 100 Hz. In self–healing property tests, fixing frequency at 1 Hz, time sweeps were employed to evaluate the self–healing capability of the hydrogels. The strain was initially set at 2% for 2.5 min, increased to 100% for 2.5 min, and then restored to 2% for an additional 2.5 min. The changes in storage modulus (G′) and loss modulus (G″) were assessed for the self–healing capacity of the samples.

Scanning Electron Microscopy (SEM). To investigate the microstructures of the composite hydrogels, SEM was employed. The hydrogel sample was freeze–dried in a vacuum extractor for 24 h by a freeze dryer. A small volume of xerogel was pasted into silica wafers. Subsequently, the samples were subjected to gold plating and observation by using a microscope.

Self–Healing Experiments. The two hydrogels were stained by methylene blue and eosin, respectively. The unstained hydrogel and two stained hydrogels are contacted in air with no other stress or outside stimulus during the healing process. Subsequently, the gap between the two stained hydrogels at different times was observed by using a microscope. All of the situations of the composite hydrogels were photographed.

Antibacterial Activity Evaluation. In vitro antibacterial activities of the hydrogels were evaluated against pathogenic representatives of Gram–positive bacteria *Staphylococcus aureus* (*S. aureus*) and Gram–negative bacteria *Escherichia coli* (*E. coli*). The hydrogels were put into bacteria culture tubes with liquid nutrient medium, which provided the bacteria with a certain concentration. The bacteria culture tubes were shocked at 37 °C for 12 h in a shaker. Then, the liquid nutrient medium was transferred onto the surface of the Luria–Bertani (LB) plates. The LB plates were incubated at 37 °C for 12 h in an incubator. The colony formation unit of LB plates was measured, which reflected the antibacterial activity of hydrogels.

## 4. Conclusions

In summary, rapid self–healing, stretchable, moldable and antibacterial composite hydrogels were successfully prepared by poly(vinyl alcohol) (PVA), borate (B), ε-poly-l-lysine (EPL), and hyaluronic acid (HA) based on the one-pot method. SEM images of the fabricated hydrogels exhibited networks with porous structures. FTIR confirmed the existence of the hydrogen bonding in the system. In addition, rheology measurements proved the dynamic properties of the networks of hydrogels. Interestingly, the fabricated hydrogels showed outstanding rapid self–healing, stretchable, and moldable performances, which could completely heal within 1 min and could be molded and remolded to various shapes. Meanwhile, the composite hydrogels displayed excellent antibacterial activity for *Staphylococcus aureus* (*S. aureus*) and *Escherichia coli* (*E. coli*). Overall, the composite dynamic network hydrogels would lay the foundation of composite hydrogels in biomedical fields, especially for wound dressing and antibacterial materials.

## Data Availability

Data are contained within the article and Supplementary Materials.

## Supplementary Materials

The following supporting information can be downloaded at: https://www.mdpi.com/article/10.3390/molecules29194666/s1. Figure S1: The photographs of the states of the PB, PBH, PBL, PBLH hydrogels at initial and after certain hours. Figure S2: Photographs of the self-healing process of PBLH hydrogels by microscope. The hydrogels were stained by methylene blue and eosin, respectively. Figure S3: The photographs of the stretchable states of the PBLH composite hydrogels.

## References

[1] Yang D. (2022). Recent Advances in Hydrogels. Chem. Mater..

[2] Wang W., Yuan Z., Li T., Wang Y., Zhang K., Wu J., Zhang S., Yuan F., Dong W. (2024). Rapid Preparation of Highly Stretchable and Fast Self-Repairing Antibacterial Hydrogels for Promoting Hemostasis and Wound Healing. ACS Appl. Bio Mater..

[3] Dehghan-Niri M., Vasheghani-Farahani E., Eslaminejad M., Tavakol M., Bagheri F. (2023). Preparation of Gum Tragacanth/Poly (vinyl alcohol)/Halloysite Hydrogel using Electron Beam Irradiation with Potential for Bone Tissue Engineering. Carbohyd. Polym..

[4] Guo W.-Y., Ma M.-G., Choi W., Kohane D.S. (2024). Hybrid Nanoparticle-Hydrogel Systems for Drug Delivery Depots and Other Biomedical Applications. ACS Nano.

[5] Toufanian S., Mohammed J., Winterhelt E., Lofts A., Dave R., Coombes B.K., Hoare T. (2024). A Nanocomposite Dynamic Covalent Cross-Linked Hydrogel Loaded with Fusidic Acid for Treating Antibiotic-Resistant Infected Wounds. ACS Appl. Bio Mater..

[6] Yin H., Liu F., Abdiryim T., Liu X. (2023). Self-Healing Hydrogels: From Synthesis to Multiple Applications. ACS Mater. Lett..

[7] Ding X., Fan L., Wang L., Zhou M., Wang Y., Zhao Y. (2023). Designing Self-healing Hydrogels for Biomedical Applications. Mater. Horiz..

[8] Rammal H., GhavamiNejad A., Erdem A., Mbeleck R., Nematollahi M., Emir Diltemiz S., Alem H., Ali Darabi M., Ertas Y., Catersonm E.J. (2021). Advances in Biomedical Applications of Self-healing Hydrogels. Mater. Chem. Front..

[9] Sun P., Ren S., Liu F., Wu A., Sun N., Shi L., Zheng L. (2018). Smart Low Molecular Weight Hydrogels with Dynamic Covalent Skeletons. Soft Matter.

[10] Luo C., Guo A., Zhao Y., Sun X. (2022). A High Strength, Low Friction, and Biocompatible Hydrogel from PVA, Chitosan and Sodium Alginate for Articular Cartilage. Carbohyd. Polym..

[11] Cho S., Hwang S., Dongyeop X., Park J. (2021). Recent Progress in Self-healing Polymers and Hydrogels based on Reversible Dynamic B-O Bonds: Boronic/Boronate Esters, Borax, and Benzoxaborole. J. Mater. Chem. A.

[12] Shui T., Pan M., Li A., Fan H., Wu J., Liu Q., Zeng H. (2022). Poly (vinyl Alcohol) (PVA)-Based Hydrogel Scaffold with Isotropic Ultratoughness Enabled by Dynamic Amine-Catechol Interactions. Chem. Mater..

[13] Yang X., Wang B., Sha D., Liu Y., Liu Z., Shi K., Liu W., Yu C., Ji X. (2021). PVA/Poly(hexamethylene guanidine)/Gallic Acid Composite Hydrogel Films and Their Antibacterial Performance. ACS Appl. Polym. Mater..

[14] Zhang X., Ren K., Xiao C., Chen X. (2023). Guanosine-driven Hyaluronic Acid-based Supramolecular Hydrogels with Peroxidase-like Activity for Chronic Diabetic Wound Treatment. Acta Biomater..

[15] Guo H., Huang S., Xu A., Xue W. (2022). Injectable Adhesive Self-Healing Multiple-Dynamic-Bond Crosslinked Hydrogel with Photothermal Antibacterial Activity for Infected Wound Healing. Chem. Mater..

[16] Li Q., Zhang S., Du R., Yang Y., Liu Y., Wan Z., Yang X. (2023). Injectable Self-Healing Adhesive Natural Glycyrrhizic Acid Bioactive Hydrogel for Bacteria-Infected Wound Healing. ACS Appl. Mater. Interfaces.

[17] Zhang Z., Wang X., Wang Y., Hao J. (2018). Rapid-Forming and Self-Healing Agarose-Based Hydrogels for Tissue Adhesives and Potential Wound Dressings. Biomacromolecules.

[18] Karimi A., Khodadadi A. (2016). Mechanically Robust 3D Nanostructure Chitosan-Based Hydrogels with Autonomic Self-Healing Properties. ACS Appl. Mater. Interfaces.

[19] Fan L., He Z., Peng X., Xie J., Su F., Wei D.-X., Zheng Y., Yao D. (2021). Injectable, Intrinsically Antibacterial Conductive Hydrogels with Self-Healing and pH Stimulus Responsiveness for Epidermal Sensors and Wound Healing. ACS Appl. Mater. Interfaces.

[20] Yan B., Huang J., Han L., Gong L., Li L., Israelachvili J.N., Zeng H. (2017). Duplicating Dynamic Strain-Stiffening Behavior and Nanomechanics of Biological Tissues in a Synthetic Self-Healing Flexible Network Hydrogel. ACS Nano.

[21] Li P., Zong H., Li G., Shi Z., Yu X., Zhang K., Xia P., Yan S., Yin J. (2023). Building a Poly(amino acid)/Chitosan-Based Self-Healing Hydrogel via Host-Guest Interaction for Cartilage Regeneration. ACS Biomater. Sci. Eng..

[22] Narasimhan B., Dixon A.W., Mansel B., Taberner A., Mata J., Malmström J. (2023). Hydrogen Bonding Dissipating Hydrogels: The Influence of Network Structure Design on Structure-Property Relationships. J. Colloid Interface Sci..

[23] Wang L., Gao G., Zhou Y., Xu T., Chen J., Wang R., Zhang R., Fu J. (2019). Tough, Adhesive, Self-Healable, and Transparent Ionically Conductive Zwitterionic Nanocomposite Hydrogels as Skin Strain Sensors. ACS Appl. Mater. Interfaces.

[24] Xu X., Victor Jerca V., Hoogenboom R. (2021). Bioinspired Double Network Hydrogels: From Covalent Double Network Hydrogels via Hybrid Double Network Hydrogels to Physical Double Network Hydrogels. Mater. Horiz..

[25] Rodell C.B., Dusaj N.N., Highley C.B., Burdick J.A. (2016). Injectable and Cytocompatible Tough Double-Network Hydrogels through Tandem Supramolecular and Covalent Crosslinking. Adv. Mater..

[26] Yin Y., Gu Q., Liu X., Liu F., Julian McClement D. (2023). Double Network Hydrogels: Design, Fabrication, and Application in Biomedicines and Foods. Adv. Colloid Interface Sci..

[27] Ollier R.C., Xiang Y., Yacovelli A.M., Webber M.J. (2023). Biomimetic Strain-Stiffening in Fully Synthetic Dynamic-Covalent Hydrogel Networks. Chem. Sci..

[28] Rezanejade Bardajee G., Ghadimkhani R., Jafarpour F. (2024). A Biocompatible Double Network Hydrogel based on Poly (acrylic acid) Grafted onto Sodium Alginate for Doxorubicin Hydrochloride Anticancer Drug Release. Int. J. Biol. Macromol..

[29] Gong J., Katsuyama Y., Kurokawa T., Osada Y. (2003). Double-Network Hydrogels with Extremely High Mechanical Strength. Adv. Mater..

[30] Nakajima T., Fukuda Y., Kurokawa T., Sakai T., Chung U.-I., Gong J. (2013). Synthesis and Fracture Process Analysis of Double Network Hydrogels with a Well-Defined First Network. ACS Macro Lett..

[31] Baumberger T., Caroli C., Martina D. (2006). Solvent Control of Crack Dynamics in a Reversible Hydrogel. Nat. Mater..

[32] Wang Y., Zhang Y., Ren P., Yu S., Cui P., Nielsen C.B., Abrahams I., Briscoe J., Lu Y. (2024). Versatile and Recyclable Double-Network PVA/Cellulose Hydrogels for Strain Sensors and Triboelectric Nanogenerators under Harsh Conditions. Nano Energy.

[33] Li L., Wu P., Yu F., Ma J. (2022). Double Network Hydrogels for Energy/Environmental Applications: Challenges and Opportunities. J. Mater. Chem. A.

[34] Chen W.-P., Hao D.-Z., Hao W.-J., Guo X.-L., Jiang L. (2018). Hydrogel with Ultrafast Self-Healing Property Both in Air and Underwater. ACS Appl. Mater. Interfaces.

[35] Ge W., Cao S., Shen F., Wang Y., Ren J., Wang X. (2019). Rapid Self-healing, Stretchable, Moldable, Antioxidant and Antibacterial Tannic Acid-cellulose Nanofibril Composite Hydrogels. Carbohyd. Polym..

[36] Teng J., Zhao W., Zhang S., Yang D., Liu Y., Huang R., Ma Y., Jiang L., Wei H., Zhang J. (2024). Injectable Nanoparticle-Crosslinked Xyloglucan/ε-Poly-l-lysine Composite Hydrogel with Hemostatic, Antimicrobial, and Angiogenic Properties for Infected Wound Healing. Carbohyd. Polym..

[37] Xu Q., Dai X., Yang L., Liu X., Li Y., Gao F. (2022). ε Polylysine-Based Macromolecules with Catalase-Like Activity to Accelerate Wound Healing by Clearing Bacteria and Attenuating Inflammatory Response. ACS Biomater. Sci. Eng..

[38] Lee J.-H., Kwon H.-K., Shin H.-J., Nam G.-H., Kim J.-H., Cho S. (2018). Quasi-Stem Cells Derived from Human Somatic Cells by Chemically Modified Carbon Nanotubes. ACS Appl. Mater. Interfaces.

[39] Meng Z., He Y., Wang F., Hang R., Zhang X., Huang X., Yao X. (2021). Enhancement of Antibacterial and Mechanical Properties of Photocurable ε Poly L lysine Hydrogels by Tannic Acid Treatment. ACS Appl. Bio Mater..

[40] Wei P., Duan Y., Wang C., Sun P., Sun N. (2024). Co-Assembled Supramolecular Organohydrogels of Amphiphilic Zwitterion and Polyoxometalate with Controlled Microstructures. Molecules.

[41] Zhang X., Xu J., Lang C., Qiao S., An G., Fan X., Zhao L., Hou C., Liu J. (2017). Enzyme-Regulated Fast Self-Healing of a Pillararene-Based Hydrogel. Biomacromolecules.

[42] Huang J., Wu C., Yu X., Li H., Ding S., Zhang W. (2021). Biocompatible Autonomic Self-healing PVA-TA Hydrogel with High Mechanical Strength. Macromol. Chem. Phys..

[43] Si R., Wang Y., Yang Y., Javeed A., Chen J., Han B. (2023). Dynamic Dual-Crosslinking Antibacterial Hydrogel with Enhanced Bio-adhesion and Self-healing Activities for Rapid Hemostasis in Vitro and in Vivo. Mater. Des..

[44] Peng Y.-Y., Cheng Q., Wang W., Wu M., Diaz-Dussan D., Kumarc P., Narain R. (2021). Multi-Responsive, Injectable, and Self-healing Hydrogels based on Benzoxaborole-Tannic Acid Complexation. Polym. Chem..

[45] Yang X., Liu G., Peng L., Guo J., Tao L., Yuan J., Chang C., Wei Y., Zhang L. (2017). Highly Efficient Self-Healable and Dual Responsive Cellulose-Based Hydrogels for Controlled Release and 3D Cell Culture. Adv. Funct. Mater..

