# Supplementary material for: Composite Hydrogels with Rapid Self-Healing, Stretchable, Moldable and Antibacterial Properties Based on PVA/ε-Poly-l-lysine/Hyaluronic Acid
Source: Molecules. 2024 Sep 30;29(19):4666. doi: 10.3390/molecules29194666 (PMC11477695; doi:10.3390/molecules29194666)
Supplement: Supplementary file 1 [file molecules-29-04666-s001.zip › molecules-3213407-supplementary.pdf]

Supplementary Materials

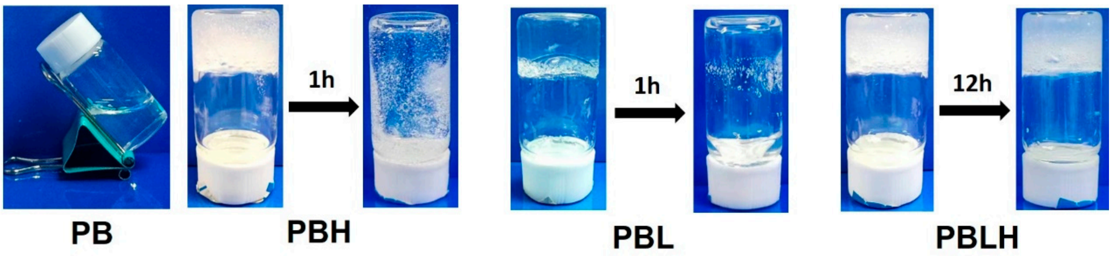

Figure S1. The photographs of the states of the PB, PBH, PBL, PBLH hydrogels at initial and after certain hours.

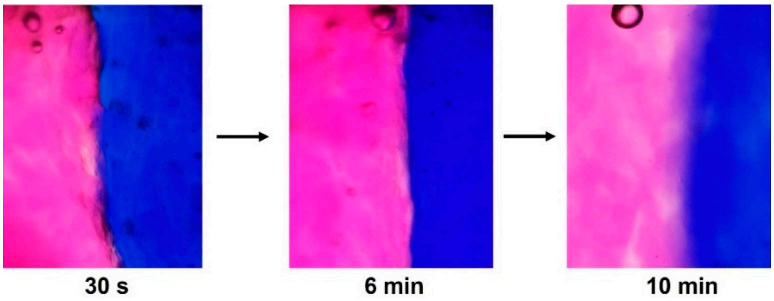

Figure S2. Photographs of the self-healing process of PBLH hydrogels by microscope. The hydrogels were stained by methylene blue and eosin, respectively.

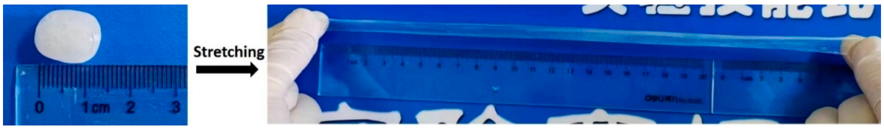

Figure S3. The photographs of the stretchable states of the PBLH composite hydrogels.
